# Easy-to-Use InDel Markers for Genetic Mapping between Col-0 and L*er*-0 Accessions of *Arabidopsis thaliana*

**DOI:** 10.3390/plants9060779

**Published:** 2020-06-22

**Authors:** Takahiro Tanaka, Yuichi Nishii, Hirotoshi Matsuo, Taku Takahashi

**Affiliations:** Graduate School of Natural Science and Technology, Okayama University, Okayama 700-8530, Japan; pwkm6m8a@s.okayama-u.ac.jp (T.T.); ptkg9kuh@s.okayama-u.ac.jp (Y.N.); peu91tyx@s.okayama-u.ac.jp (H.M.)

**Keywords:** InDel markers, SSLP, chromosome mapping, *Arabidopsis thaliana*, mutants

## Abstract

Map-based gene cloning has played a key role in many genetic studies using the model plant, *Arabidopsis thaliana*. In the post- next generation sequencing era, identification of point mutations and their corresponding genes is increasingly becoming a powerful and important approach to define plant gene function. To perform initial mapping experiments efficiently on *Arabidopsis* mutants, enrichment of easy-to-use and reliable polymorphic DNA markers would be desirable. We present here a list of InDel polymorphic markers between Col-0 and L*er*-0 accessions that can be detected in standard agarose gel electrophoresis.

## 1. Introduction

In the last decades, many genes have been identified from mutations by map-based cloning strategy in the model plant, *Arabidopsis thaliana* [1,2]. Even after the development of genetic engineering techniques such as CRISPR/Cas, which has proven to be a powerful tool for gene editing [3,4,5,6], isolation of point mutant alleles and identification of their corresponding genes still continue to be an indispensable approach to the study of gene function. With the advance of PCR technology, there have been various mapping tools and markers developed. These include cleaved amplified polymorphic sequences (CAPS) [7,8], simple sequence length polymorphisms (SSLP) also known as microsatellites [9] and derived CAPS (dCAPS) [10]. In the post-next generation sequencing (NGS) era, chromosome mapping with the use of these polymorphic markers remains an initial key step for rapid detection and identification of a mutant gene. This is both because the mutants isolated by chemically induced mutagenesis contain multiple mutations and because NGS data contains many sequencing errors.

To perform mapping experiments efficiently, insertion/deletion (InDel) markers including SSLP, which can discriminate genotypes without restriction digestion, have been further developed between *Arabidopsis* accessions [11,12] and, indeed, widely applied to many genetic studies. Information on these polymorphic markers is also available in the TAIR database (http://www.arabidopsis.org). However, it may not be simple to choose the markers appropriate for initial mapping experiments from the vast information compiled from many sources, which sometimes also contains incorrect data. One of the few disadvantages of these InDel markers is that most of them represent length polymorphisms of less than 30–40 bp and require electrophoresis in polyacrylamide or highly concentrated agarose gels for detection. On the other hand, the number of the markers with more length difference detectable in standard 0.8%–1.0% agarose gels is limited. A study by Zhang et al. [13] provides a useful list of InDel markers between Columbia-0 (Col-0) and Landsberg *erecta*-0 (L*er*-0), the most frequently used accessions in *Arabidopsis* genetic studies, with a detailed protocol for map-based cloning. The list contains two InDel per chromosome for a total of ten markers. In order to save time and effort from initial rough mapping to final gene identification, further InDel markers would be desirable. Here, based on published papers and the information collected in the database, we provide a list of InDel markers between Col-0 and L*er*-0 accessions that can be detected in standard agarose gel electrophoresis.

## 2. Results and Discussion

**Figure 1:**
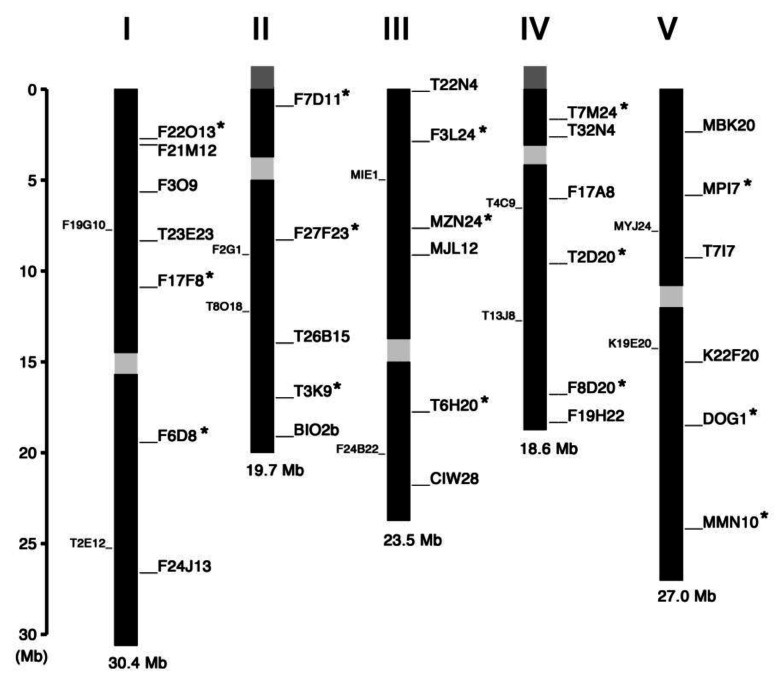
Linkage map of the InDel markers on *Arabidopsis* chromosomes. The markers examined in this study are indicated in the right-hand side of each chromosome whose number is shown on the top. The markers recommended as a first choice for rough mapping are asterisked. The InDel markers reported by Zhang et al. [13] are indicated in the left-hand side of each chromosome. The centromere regions are shown in light gray boxes. Nucleolus organizer regions coding for ribosomal RNAs are shown in dark gray boxes at the end of upper arm of chromosomes II and IV.

We searched for and selected InDel polymorphisms with length difference of more than 60 bp between Col-0 and L*er*-0 accessions, which would be separable on 0.8% agarose gels, from the information available in the TAIR database (http://www.arabidopsis.org) and previously published papers [11,12,13,14,15]. Figure 1 summarizes the chromosome map position of all markers we have tested so far and judged to be appropriate for genotyping. The name of these markers is derived from the name of bacterial artificial chromosome (BAC) clones in which the InDel polymorphism exists, except for BIO2b, CIW28 and DOG1 [15]. We recommend the markers asterisked in Figure 1 as a first choice for initial rough mapping because they are evenly distributed across the chromosomes and both upper and lower arms of each chromosome hosted at least one of them.

With only a few exceptions, we designed PCR primers to be 20 nucleotides in length with the GC content of 9 to 11 nucleotides and the 3′ ending with G or C to promote specific binding (Table 1). To prevent mispriming at GC-rich sequences and smearing of DNA bands in the gel, A or T was placed at the second position from the 3′ end and inclusion of more than 3 consecutive G/Cs near the 3′ end was avoided [16]. The length of amplified DNA fragments was generally set ranging from 300 bp to 1000 bp.

**Figure 2:**
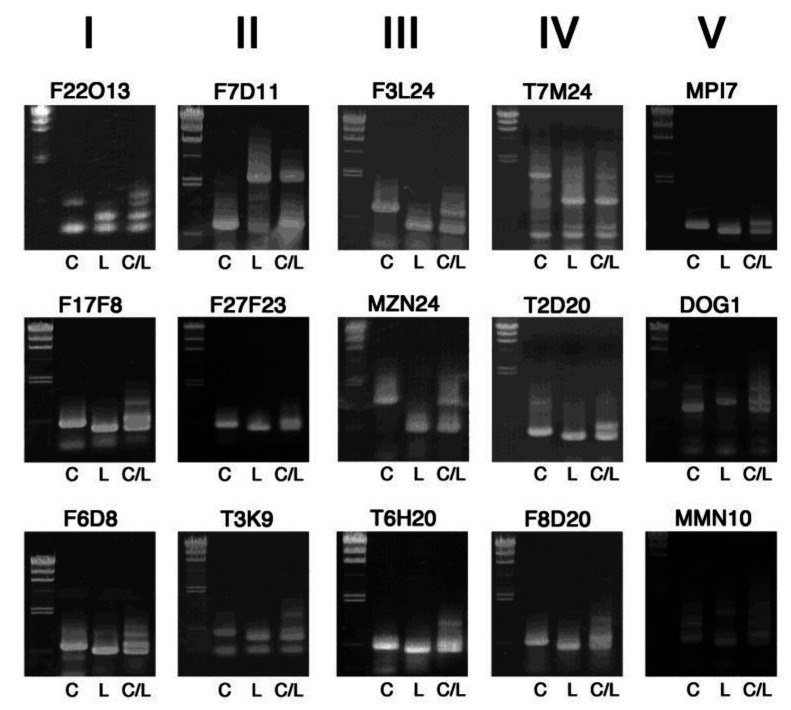
Band patterns of InDel markers useful for initial rough mapping. PCR-amplified DNA fragments were electrophoresed on 0.8% agarose gels, together with size markers of lambda DNA digested with *Hin*dIII (23.1, 9.4, 6.6, 4.4, 2.3, 2.0 and 0.56 kb) on the left side. C, L and C/L indicate template DNA from Col-0, L*er*-0 and their F1 heterozygous progeny, respectively. The chromosome number is shown on the top.

Figure 2 shows PCR-amplified fragments of the first recommended 15 markers in 0.8% agarose gels, which are arranged by the chromosome number (I–V) and position of each InDel marker. These markers exhibit clear difference in length.

**Figure 3:**
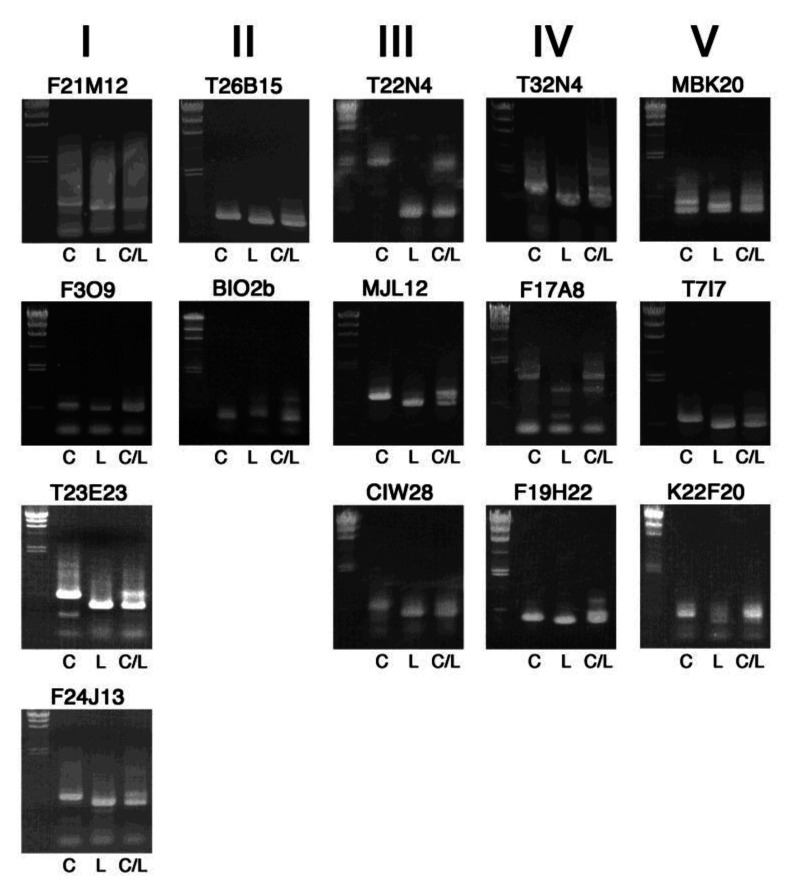
Band patterns of InDel markers for further mapping experiments. PCR-amplified DNA fragments were electrophoresed on 0.8% agarose gels, together with size markers of lambda DNA digested with *Hin*dIII (23.1, 9.4, 6.6, 4.4, 2.3, 2.0 and 0.56 kb) on the left side. C, L and C/L indicate template DNA from Col-0, L*er*-0 and their F1 heterozygous progeny, respectively. The chromosome number is shown on the top.

Band patterns of PCR products of additional 15 markers, arranged by their chromosome number (I–V) and position, are shown in Figure 3. These are also clear enough for genotyping in 0.8% agarose gels, which we routinely use. Although extra DNA bands are found in some markers, this may be further improved by modifying PCR conditions. The use of 1.0% or 1.2% gels would be better for higher resolution of the DNA fragments, in particular, with length less than 500 bp.

The list shown in this study is not exhaustive. A detailed study of the L*er*-0 genome revealed that more than one hundred single-copy genes are only present in the reference sequence or the L*er*-0 assembly [17]. This clearly suggests that the InDel markers suitable for fine mapping can be further developed. Together with all the other known polymorphic markers, the use of these InDel markers should make mapping experiments easier and accelerate functional analysis of genes with their point mutant alleles in *Arabidopsis*. Although we focused only on the most popular accessions Col-0 and L*er*-0 in this work, some studies have identified mutants in Niederzenz-0 (Nd-0) [18] or other natural accessions. Some of the markers presented here are also expected to be applied to mapping experiments with these accessions.

## 3. Materials and Methods

### 3.1. DNA Extraction

Genomic DNA was extracted from a leaf or a seedling of Col-0, L***er***-0 and their F1 progeny plants. The sample was ground with micro pestle in a 1.5-mL microtube for 30 s. After addition of 120 µL DNA extraction buffer that contains 0.2 M Tris-HCl (pH 8.0), 0.25 M NaCl, 25 mM EDTA and 0.5% SDS (w/v), the microtube was vortexed for 15 s and centrifuged at 15,000 rpm at room temperature for 10 min. The supernatant of 100 µL was transferred to another tube, mixed with 100 µL isopropanol, and precipitated for 10 min in a freezer. DNA was collected by centrifugation at 12,000 *g* for 10 min, rinsed with 100 µL of 70% ethanol, dried under vacuum for 10 min and dissolved in 40 µL TE buffer. After centrifugation at 12,000 *g* for 1 min, the supernatant was used as a template DNA for PCR.

### 3.2. PCR and Agarose Gel Electrophoresis

PCR was performed using ExTaq polymerase (Takara, Kyoto, Japan) with 45 cycles of denaturation at 94 °C for 30 s, annealing at 55 °C for 30 s and extension at 72 °C for 90 s. The PCR products were separated on 0.8% (w/v) agarose gels in TAE buffer and visualized with ethidium bromide.

## Figures and Tables

**Table 1:** PCR primer pair sequences used to examine the 30 InDel markers of this study.

Marker Name	Chr.	Position (Mbp)	Gene ID *	Length (bp) (Col-0/Ler)	Primer Sequence
F22O13	1	2.826	At1g04667	460/210	F: GTGTTGGGGAGAGCTTATAG
					R: TCCACTTCCAACCATCAGAG
F21M12	1	3.138	At1g09700 (*HYL1*)	580/500	F: AAGACTCCATCTTGACACTG
					R: CCTCAACCTACTGATCATTG
F3O9	1	5.653	At1g16530 (*ASL9*)	610/530	F: TTTTGGTCGGGTATGGAATG
					R: CCAGAAGTTGCTCGTTAAAG
T23E23	1	8.494	At1g24000	540/420	F: AAGGTCTTGTAGCGATCTAG
					R: AACCCAACTGGCTCATTTTG
F17F8	1	11.016	At1g30930	460/360	F: GGAAGAGGATTGACTCAAAG
					R: CTACCGCTAGGACTTTCATG
F6D8	1	19.621	At1g52690 (*LEA7*)	640/540	F: GAGACACAGAGGAAGTGAAG
					R: CTGACCAGCAAATTCTCAAG
F24J13	1	26.624	At1g70610 (*ABCB26*)	600/500	F: GCTACCCTTCAAGAGATGAG
					R: TCGTAGAGTTGCAGCAAAAG
F7D11	2	1.614	At2g04622	670/580	F: AGCGAACTTCGTTGATGTTC
					R: CAATGTATATGCTCTTCTAGAG
F27F23	2	8.410	At2g19410	460/400	F: TGACCAGTTGTACCAATGTG
					R: GTCTGCGACAAAAAATACTG
T26B15	2	13.825	At2g32560	470/410	F: AACACACTCTCTCTCTCTTG
					R: AGGTCAAGAACCGACATTTG
T3K9	2	17.107	At2g40990	470/360	F: TCATCGGAAGGAGCATTATG
					R: AGGATGTTCCAGAGAGAATG
BIO2b	2	18.012	At2g43360 (*BIO2*)	390/460	F: TGTACCTCCCTGAAGTTATG
					R: TCTTGACCTCCTCTTCCATG
T22N4	3	0.130	At3g01345	2040/400	F: TGACTGTTTGACTCCAAGTG
					R: GTTACGAACCTCTGGTATTG
F3L24	3	2.849	At3g09270 (*GSTU8*)	770/470	F: TGAGCAATGATGGTTAGCAG
					R: GAACGTAACTGCTTACGTAG
MZN24	3	7.665	At3g21750 (*UGT71B1*)	1250/500	F: ATCCGAACCGAAATCAACTG
					R: GACTGAACGAGAGGAACATG
MJL12	3	9.194	At3g25240	650/490	F: GGAGGCTAGAGACTCATATG
					R: AGGGGATATTCGACTGAGAG
T6H20	3	17.243	At3g46820 (*TOPP5*)	510/430	F: AGTCCACCATGCATACAAAG
					R: TGCATTGGTTTCTCTGCTTG
CIW28	3	21.869	At3g59140 (*ABCC10*)	630/450	F: GAGCACAAGTCTCTTACAAG
					R: CCCTAAGTTTCACAAAGAATG
T7M24	4	1.788	At4g03826	1350/710	F: TTTGGCGCTGTTGCCAATTG
					R: TAATGCGCGAGGTGGATATG
T32N4	4	2.549	At4g04985	1180/900	F: CTCAAGGTCGACATGATAATG
					R: GTATAACGCGGGTCAATCTC
F17A8	4	6.109	At4g09670	1280/560	F: TGCTCGAGAGACTTTTCGAG
					R: CATAGACAGCCACACCAATG
T2D20	4	9.652	At4g17200	420/360	F: TGGTCTTCTTATGCTCCAAG
					R: AGAGGAAGCACACAGTATTG
F8D20	4	16.924	At4g35700 (*DAZ3*)	540/460	F: GGCGAGGATTGACTTAAATG
					R: ACTGTTGCGATAATGCAGTG
F19H22	4	18.133	At4g38870	380/320	F: GCGTTGTTGAGTGTAGCAAG
					R: GAGATCGATCGTCATCTTTC
MBK20	5	2.476	At5g07770 (*FH16*)	410/350	F: AGAGACCCTTTTCTCTGTTG
					R: GGAGCTTACCATCATATCAG
MPI7	5	5.902	At5g17860 (*CAX7*)	570/490	F: TCCAATTAGACCGCATATTAG
					R: TTCGTTGCTTGAGACACTAG
T7I7	5	9.271	At5g26594 (*RR24*)	650/500	F: TGGCACCAAGAAGCAACTAG
					R: TCCTAACTATCAACCAACTTG
K22F20	5	15.027	At5g00540	490/420	F: ACCGCTACCATTTGTTCTTG
					R: CCAACGTTCTTCCCTGTTAG
DOG1	5	18.591	At5g45830 (*DOG1*)	1010/1300	F: GCGTGTTTGTGTTTTGTGTG
					R: ATCCGCTGTCTCAGGACATC
MMN10	5	24.108	At5g59840 (*RABE1B*)	530/460	F: TGAAGGATGACTCGTCTGTG
					R: GATGGCTCTTTCACCACTAG

* Gene ID represents a gene that shows or is closest to the InDel polymorphism.
